# 8q12 microduplication including CHD7: clinical report on a new patient with Duane retraction syndrome type 3

**DOI:** 10.1186/1755-8166-6-49

**Published:** 2013-11-08

**Authors:** Anna Baroncini, Sara Bertuzzo, Rita Quarantini, Paolo Ricciardelli, Roberto Giorda, Maria Clara Bonaglia

**Affiliations:** 1 Medical Genetics Unit, Maternal and Child health Department, ASL of Imola, Imola, Italy; 2 Cytogenetics Laboratory, Scientific Institute, IRCCS Eugenio Medea, Bosisio Parini, Via Don Luigi Monza, 20, 23842 Bosisio Parini(LC), Italy; 3 Child and Adolescent Neuropsychiatric Unit, Mental Health and Pathological Dependencies Department, ASL of Ravenna, Ravenna, Italy; 4 Pediatric Unit, Maternal and Child health Department, ASL of Ravenna, Ravenna, Italy; 5 Molecular Biology Laboratory, Scientific Institute, IRCCS Eugenio Medea, Bosisio Parini, Lecco, Italy

**Keywords:** 8q12 microduplication, Array-CGH, CHD7, Duane retraction syndrome

## Abstract

**Background:**

A novel multiple congenital anomalies syndrome has been recently identified in four patients carrying a 8q12 microduplication sharing the smallest region of overlap (SRO, size 1.6 Mb) including five genes *CA8, ASPH, RAB2B, CLVS1 and CDH7.* The phenotype is mainly characterized by neurodevelopmental delay, heart defects, facial features and Type 1 Duane anomaly. Increasing dosage of *CDH7* was proposed to be responsible for the recurrent pattern of MCA.

**Results:**

High resolution array-CGH analysis identified a 4.2 Mb *de novo* interstitial duplication of the 8q12.1-q12.3 chromosome region in a boy with developmental delay, dysmorphic features, type 3 Duane anomaly. This duplication includes several genes and spans the SRO.

**Discussion:**

The present case represents a further patient with an interstitial duplication of chromosome 8q12 and several shared clinical features. Although more cases are needed to delineate the full-blown phenotype of 8q12 duplication syndrome, published data and present observations suggest that it results in a clinically recognizable phenotype. The presence of Duane anomaly in four out of five described patients with a 8q12 duplication definitely rules against the possibility of its being a chance finding unrelated to the imbalance and points toward a pathogenic role. Gene content analysis of the duplicated region and review of the literature suggest that gain-of-dosage of the *CHD7* gene may be a good candidate for the main clinical features of the syndrome.

## Background

Microduplication of 8q12, encompassing the *CHD7* gene, which is mutated or deleted in CHARGE syndrome, has recently been identified to result in a novel multiple congenital anomalies syndrome. Hitherto four patients have been described, with duplication sizes ranging from 2.7 to 6.9 Mb and different breakpoints within the 8q12 region [1-4]. The phenotype, though variable, is mainly characterized by neurodevelopmental delay, heart defects, and facial features. Type 1 Duane anomaly has been reported in three out of the four described cases, the exception being the child with the smaller duplication. Duane retraction syndrome (DRS) is a highly heterogeneous eye-movement disorder that in a quarter to half of cases is associated with additional congenital defects and/or genetic syndromes [5]. Beyond the present microduplication, involvement of chromosome 8 with Duane anomaly has been documented in mosaic trisomy 8 [6,7], in 8q13 deletions [8,9], and in a de novo reciprocal balanced translocation disrupting the *CPA6* gene in 8q13 where the Duane retraction syndrome 1 locus (DURS1) is located [10].

Here we report on a further child who carries a de novo 4.2 Mb duplication of the region 8q12.1-q12.3 presenting with developmental delay, dysmorphic features, and type 3 Duane anomaly in order to refine the clinical presentation of the 8q12 microduplication syndrome and to contribute to genotype-phenotype correlation.

## Case presentation

The child was referred to our Clinical Genetics Service for dysmorphic features and developmental delay. The patient was the third child of healthy unrelated parents with uneventful family history. He was born at 39 weeks of gestation, after a normal pregnancy, with urgent caesarean section due to placental abruption. No intrauterine exposure to drugs and other possibly harmful factors were reported. At birth, weight was 2570 gr, length 47 cm, head circumference 32 cm, and thoracic circumference 33 cm. APGAR scores were 9/10 at 1’/5’ , respectively. At cardiological evaluation, a 2/6 systolic ejection murmur was detected and an echocardiogram showed a patent foramen ovale with left-to-right shunt, which spontaneously disappeared within six months of life. Developmental delay and muscle hypotonia were observed since the first months of life.

**Figure 1:**
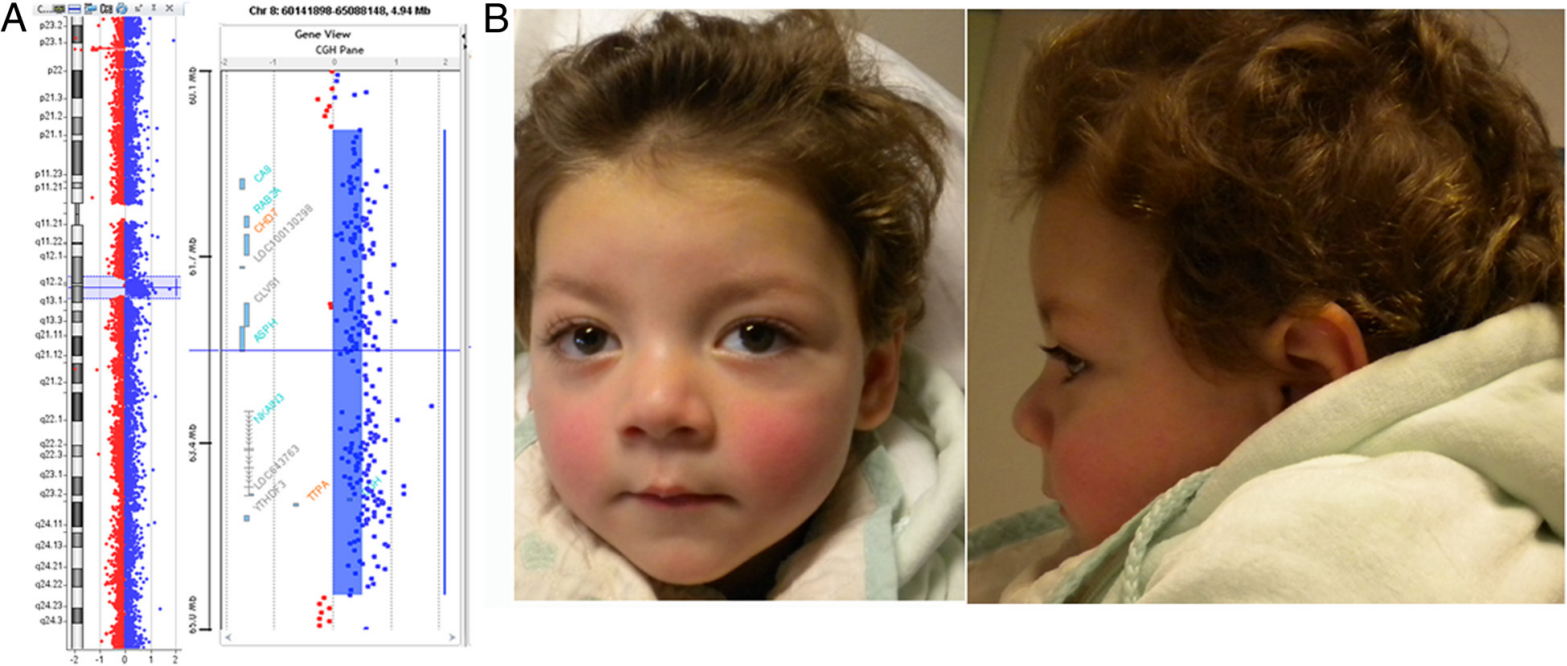
**Molecular details and facial appearance of the patient. (A)** Array-CGH results. (left), array CGH profile of chromosome 8 showing a duplication at 8q12.1q12.3; (right), enlargement of the 8q12.1q12.3 duplicated region. Some of the genes included in the duplicated region (from 60,667 Mb to 64,709 Mb) are listed in grey, light blue, and orange. **(B)** Photograph of the patient at the age of 16 months.

On first examination at the Genetic Clinic, at age 16 months, he was 82 cm tall (75^th^ centile), weighed 11.3 Kg (50^th^ centile) and his head circumference was 45.2 cm (< 3^rd^ centile). He had brachycephaly (cephalic index: 87%), full cheeks, medial flaring of eyebrows, long eyelashes, epicanthic folds, strabismus, normal ears with total length of 4.7 cm (50^th^ centile), short nose, low and widened nasal root, long philtrum, full lower lip, tooth enamel erosions. Chest circumference was 52 cm (75^th^ percentile) and the internipple distance was 13 cm (97^th^ percentile). Hands had normal length (50^th^-75^th^ percentile), tapering fingers and low set thumbs. Feet length was 13 cm (75^th^ percentile) (Figure 1B).

At age 28 months, stature and weight were respectively at 97^th^ and 75^th^ percentile while head circumference was still < 3^rd^ percentile.

Motor skills were significantly delayed: he was able to sit unassisted and to stand up without support since age 24 and 38 months, respectively, and began walking with assistance since age 45 months. Language delay was significant: at age 3 years and 9 months he vocalized but didn’t yet use any word to communicate. At the same age he had not yet achieved toilet training and head, trunk and hand stereotypies, noted since the first year of life, were still present though less common. Constipation and feeding problems, mainly consisting in long feeding times, difficulty chewing and fluids dysphagia, have been moderately ameliorating over the years.

A MRI of the brain, at age 7 months, showed no abnormalities except for a thin corpus callosum. Abdominal ultrasound was normal. Ear, nose and throat examination didn’t disclose anomalies and brainstem auditory evoked potentials were within normal limits. Ophthalmological evaluation, at age 3 years, detected anisocoria (R > L), slight inconstant nystagmus and exotropia in primary position of the left eye; fundoscopy was normal. Examination of the ocular motor system revealed in the left eye restricted abduction and absent adduction with globe retraction and palpebral fissure narrowing at attempted adduction and concluded for a unilateral type 3 Duane syndrome. Conventional cytogenetic studies showed a normal karyotype (46,XY).

## Results

In the absence of any etiological diagnosis, array CGH analysis using the 180 K array platform was performed in the patient detecting a 8q12.1q12.3 duplication of 4.2 Mb, as described in Figure 1A. Quantitative PCR performed on the patient and his parents evidenced that the 8q12.1q12.3duplication originated de novo, while genotyping of polymorphic loci demonstrated the paternal origin of the duplication (data not shown).

FISH analysis on metaphases from both proband’s parents excluded the presence of insertional translocations of 8q12.1q12.3 (data not shown). The final interpretation of the rearrangement was arr[hg19] 8q12.1q12.3(60,637,619×2,60,667,866-64,709,770×3,64,734,723×2)dn.

## Discussion

With this case, five patients with partially overlapping 8q12 microduplications have been described, sharing common features the most consistent of which, observed in at least four subjects, are neonatal hypotonia, developmental delay, facial features, heart defects, and Duane anomaly. A similar facial gestalt is noticed, with full cheeks or round face, flared medial eyebrows, short nose with widened root, long philtrum, full lower lip. Hearing loss, urinary malformations, and microbrachycephaly appear inconstant findings, while variable behavioural anomalies, feeding problems and constipation are present in the majority of cases. The smallest region of overlap (SRO) between the published cases, including the present one, is around 1.6 Mb and spans five genes: *CA8*, *ASPH*, *RAB2B*, *CLVS1*, and *CDH7*. Up to now, only two of these genes, *CHD7* and *CA8*, have been linked to a human disease. *CHD7* haploinsufficiency, either by mutation or deletion, is causative of the CHARGE syndrome. Homozygous mutation in the *CA8* gene, encoding carbonic anhydrase VIII, has recently been found in Congenital ataxia with predisposition to quadrupedal gait (OMIM #613227) [11].

The reported association of 8q12 microduplication and DRS suggests a possible causal involvement of the dosage-sensitive *CHD7* gene [1-3].

In fact, in all patients the *CHD7* gene is included in the duplicated region but none of them has the distinctive face nor fulfils the clinical criteria for typical or partial/incomplete Charge syndrome according to Verloes’ categorization [12] and only in the child described by Monfort et al. [1] a major sign of the syndrome, represented by a Mondini anomaly, was observed. Therefore, *CHD7* copy number increase doesn’t appear a likely pathogenic mechanism underlying Charge’s aetiology.

Duane retraction syndrome (DRS) is a congenital ocular motility disorder resulting in absent or marked restriction of abduction (type 1), adduction (type 2), or both (type 3), in addition to globe retraction and palpebral fissure narrowing on attempted adduction, as a result of a congenital anomaly of the 6th cranial nerve nuclei with paradoxical innervation of the lateral rectus from the 3rd cranial nerve. According to pooled data, type 1 is by far the most common (78%), followed by type 3 (15%) and 2 (7%). Approximately 0,1% of the general population, without ethnic preferences, and 1-4% of all strabismus cases show DRS, with a predilection for unilateral involvement of the left eye and for female patients [13].

Most cases are sporadic but familial transmission, commonly autosomal dominant, has been documented in several instances, usually in relation to isolated Duane anomaly. Beside a number of recognizable syndromes that are monogenic in origin, Duane anomaly has also been observed in a variety of numerical and structural chromosome aberrations involving different chromosomes, locations, and sizes. However, up to now the majority of these reports have been sporadic findings that might represent chance association, whereas contribution of chromosomes 8 and 20 appears to be substantial.

DRS associated with interstitial deletion of the 20q13 region is probably due to haploinsufficiency of the *SALL4* gene, causative of the Duane-Radial Ray syndrome (OMIM #607323).

In addition to the present microduplication, involvement of chromosome 8 with Duane anomaly has been documented in mosaic trisomy 8 [6,7], and in 8q13 deletions [8,9] although the precise localisation of the deletions was not defined, since the paper was published prior to the advent of microarrays.

Furthermore, a de novo balanced translocation disrupting the *CPA6* gene, located within the DURS1 locus in 8q13, was reported in a patient exhibiting features of Duane retraction syndrome [10]. As demonstrated in zebra fish, conserved expression of *CPA6* in cartilaginous precursors posterior to the eye strongly suggests a role in the aetiology of Duane anomaly. However, its deficiency alone doesn’t affect development or function of the VI nerve in the animal model and may not be enough to cause the ocular motility disorder, requiring interaction with other genes and/or enhancer elements [14]. These data suggest that involvement of the 8q13 locus in Duane anomaly still remains uncertain, while the presence of a Duane anomaly in four out of the five described patients with a 8q12 triplication definitely rules against the possibility of a coincidence unrelated with the overlapping imbalances, as previously considered among the various hypotheses, and points toward a pathogenic role.

## Conclusion

In conclusion, our patient with 8q12 deletion of 4.2 Mb, including the 1.6 Mb SRO, supports the hypothesis that *CHD7* might be the most likely candidate for generating the main clinical features of the syndrome and influencing the appearance of Duane anomaly.

## Methods

### Array-CGH analysis

Array-CGH was performed using the Agilent Human Genome CGH Microarray Kit 180 k (Agilent Technologies Inc., Santa Clara, CA) with a resolution of ~40 Kb (Figure 1A). All nucleotide positions refer to the Human Genome, Feb 2009 Assembly (hg19). Data analysis was performed using Agilent Cytogenomics version 2.5.8.1.

### Fish analysis

FISH was performed on both parents’ metaphases by using Chr8:61591397-61780768 (hg19) SureFISH probes (Agilent Technologies, Santa Clara, CA, USA) according to the manufacturer’s protocol. This probe maps on chromosome 8 at the *CHD7* gene.

### Quantitative PCR

Quantitative PCR (qPCR) analysis was performed as in [15]. The UCSC Genome Browser (Feb 2009 assembly; http://genome.ucsc.edu/) maps and sequence were used as references. Probe location was: RT1, chr8:61653966-61654028; RT2, chr8:63979108-63979170. The sequences of all primers used are available from the authors.

### Genotyping

Genotyping of polymorphic loci in the proband and his parents was performed by amplification of loci D8S260, D8S1718, and D8S1178 with primers labelled with fluorescent probes (ABI 6-Fam, Tet, and Hex), followed by analysis on ABI 3100 Genetic Analyzer (Applied Biosystems, Foster City, CA). Locus location was verified on the UCSC database.

## Consent

Written informed consent was obtained from the patient for publication of this Case report and any accompanying images. A copy of the written consent is available for review by the Editor-in-Chief of this journal.

## Competing interests

The authors declare that they have no competing interests.

## Authors’ contributions

AB was responsible for the patient’s clinical genetics examination; SB performed FISH and chromosomal molecular assay; RG and MCB conceived and designed the molecular experiments and data analysis; PR and RQ ensured follow-up of the patient and contributed to the clinical description. MCB and AB co-wrote the paper. All authors read and approved the final manuscript.
